# Children’s microvascular traits and ambient air pollution exposure during pregnancy and early childhood: prospective evidence to elucidate the developmental origin of particle-induced disease

**DOI:** 10.1186/s12916-020-01586-x

**Published:** 2020-05-26

**Authors:** Leen J. Luyten, Yinthe Dockx, Eline B. Provost, Narjes Madhloum, Hanne Sleurs, Kristof Y. Neven, Bram G. Janssen, Hannelore Bové, Florence Debacq-Chainiaux, Nele Gerrits, Wouter Lefebvre, Michelle Plusquin, Charlotte Vanpoucke, Patrick De Boever, Tim S. Nawrot

**Affiliations:** 1 Centre for Environmental Sciences, Hasselt University, Diepenbeek, Belgium; 2 Unité de Recherche en Biologie Cellulaire (URBC) - Namur Research Institute for Life Sciences (Narilis), Namur University, Namur, Belgium; 3 Health Unit, Flemish Institute for Technological Research (VITO), Mol, Belgium; 4 Belgian Interregional Environment Agency (IRCELINE), Brussels, Belgium; 5 Department of Public Health & Primary Care, Occupational and Environmental Medicine, Leuven University, Leuven, Belgium

**Keywords:** CRAE, CRVE, Tortuosity index, Air pollution, Child health

## Abstract

**Background:**

Particulate matter exposure during *in utero* life may entail adverse health outcomes later in life. The microvasculature undergoes extensive, organ-specific prenatal maturation. A growing body of evidence shows that cardiovascular disease in adulthood is rooted in a dysfunctional fetal and perinatal development, in particular that of the microcirculation. We investigate whether prenatal or postnatal exposure to PM_2.5_ (particulate matter with a diameter ≤ 2.5 μm) or NO_2_ is related to microvascular traits in children between the age of four and six.

**Methods:**

We measured the retinal microvascular diameters, the central retinal arteriolar equivalent (CRAE) and central retinal venular equivalent (CRVE), and the vessel curvature by means of the tortuosity index (TI) in young children (mean [SD] age 4.6 [0.4] years), followed longitudinally within the ENVIR*ON*AGE birth cohort. We modeled daily prenatal and postnatal PM_2.5_ and NO_2_ exposure levels for each participant’s home address using a high-resolution spatiotemporal model.

**Results:**

An interquartile range (IQR) increase in PM_2.5_ exposure during the entire pregnancy was associated with a 3.85-μm (95% CI, 0.10 to 7.60; *p* = 0.04) widening of the CRVE and a 2.87-μm (95% CI, 0.12 to 5.62; *p* = 0.04) widening of the CRAE. For prenatal NO_2_ exposure, an IQR increase was found to widen the CRVE with 4.03 μm (95% CI, 0.44 to 7.63; *p* = 0.03) and the CRAE with 2.92 μm (95% CI, 0.29 to 5.56; *p* = 0.03). Furthermore, a higher TI score was associated with higher prenatal NO_2_ exposure. We observed a postnatal effect of short-term PM_2.5_ exposure on the CRAE and a childhood NO_2_ exposure effect on both the CRVE and CRAE.

**Conclusions:**

Our results link prenatal and postnatal air pollution exposure with changes in a child’s microvascular traits as a fundamental novel mechanism to explain the developmental origin of cardiovascular disease.

## Background

A growing body of evidence shows that particulate-induced health effects are rooted in dysfunctional fetal and perinatal development [1]. At least four lines of evidence contribute to this statement. First, during the earliest phases of life, the fetus can be exposed to particulate matter via the placenta. An ex vivo human placental perfusion model has shown that trans-placental transport is able to channel particles with a diameter smaller than 240 nm [2]. In addition, an in vivo rabbit model exposed to diesel exhaust demonstrated that particles can even be transferred into the fetal bloodstream [3]. Accordingly, in human context, it was recently found that even low concentrations of ambient particles, including black carbon, are present in detectable quantities at the fetal side of the placenta [4]. Second, in utero exposure to particulate air pollution can increase oxidative stress and inflammatory markers, which potentially leads to molecular modifications in for example placental tissue [5]. Third, both DNA damage, measured by for example DNA adducts [6], elevated levels of micronuclei [7], and DNA methylation levels [8], and epigenetic markers such as DNA methylation [9], histone modification, and miRNAs expression [10], which ultimately regulate chromatin structure or gene activity, have been associated with in utero exposure to particulate air pollution. Finally, several postnatal effects related to prenatal particulate exposure have been elaborated in recent research, for example on molecular longevity, as reflected by telomere length [11], or on clinical factors such as birth weight [12]. In turn, these effects have been linked to (markers of) disease development later in life, such as increased stiffness of the carotid artery or an increased risk of cardiovascular disease development [13, 14].

The microcirculation makes up a large part of the human vasculature and undergoes extensive, organ-specific perinatal maturation [15, 16]. Over the past years, fundus photography has increasingly been used to assess microvascular health [17]. A strong correlation exists between macrovascular and microvascular parameters in adults, which has been shown for example by Seidelmann and colleagues: in 2617 persons with the highest quartile of retinal venular diameter, contrasting the persons with the lowest venular diameter quartile, this resulted in a 2.4% higher risk of atherosclerotic events during a 10-year follow-up [18]. Therefore, changes in the microvasculature of the retina at an early age can be an indicator of cardiovascular disease later in life [19, 20]. Furthermore, particulate matter air pollution is an important risk factor for adverse cardiovascular effects later in life [21–24]. Recently, our research group described an association between short-term ambient air pollution exposure at school and an increase in retinal arteriolar diameter in 10-year-old children [25]. However, to date, no studies have considered changes in the maturation of the microvasculature in relation to prenatal exposure to particulate air pollution. By making use of the prospective ENVIRonmental influence *ON* AGEing in early life (ENVIR*ON*AGE) birth cohort, we tested the hypothesis in 4- to 6-year-olds that prenatal and postnatal particulate air pollution exposures are associated with changes in the diameter and curvature of retinal blood vessels in early childhood.

## Materials and methods

### Study population

The participants of this study are enrolled in the ongoing prospective ENVIR*ON*AGE birth cohort. Detailed information on data collection within this cohort is described in the article of Janssen and colleagues [26]. Before delivery, an initial informed consent form was signed by the mothers, and after delivery, the participants filled out a questionnaire which provided us detailed medical and lifestyle data, including their residential address(es) during pregnancy, maternal age, maternal weight and height, maternal pre-pregnancy body mass index (BMI), maternal education, and maternal smoking habits and alcohol consumption throughout pregnancy. Additionally, perinatal parameters, such as the date of birth, gestational age, sex, birth weight, and birth length, were obtained from the participant’s medical files. Maternal education was coded as low (no diploma or primary school), middle (high school diploma), and high (college or university degree). Smoking status was classified as non-smokers, stopped smoking before pregnancy, and current smokers (smoked during pregnancy). Alcohol use was subdivided in mothers who did not consume alcohol during pregnancy and mothers who consumed alcohol at least occasionally during gestation.

The follow-up examinations in this study population were performed between October 3, 2014, and July 12, 2018. Within this timeframe, 587 children were between 4 and 6 years old and could hence participate in the first follow-up step of ENVIR*ON*AGE. Thirteen mother-child pairs were not eligible for participation since their child had passed away (*n* = 1) or they had moved to another country or too far from the location where the examination took place (*n* = 12). Of the remaining 574 mother-child pairs, 74 people could not be contacted by e-mail or phone since no up-to-date contact details could be retrieved, three mother-child pairs could not be contacted at the moment that the child had an age between four and six, 165 women refused to participate, and 332 renewed consent (i.e., a final participation rate of 58%) (Additional file 1: Fig. S1). A second questionnaire was filled out by the participants on the day of the follow-up examination, which provided us with information on lifestyle conditions of the child, for example on the passive exposure to parental smoking. Passive smoking status was classified as not exposed, exposed by one parent, or exposed via smoking of both parents.

### Clinical measurements

The clinical investigation of the retinal blood vessels and blood pressure was performed by a single trained examiner, following standardized guidelines. Children always gave their assent before measurements were initiated. The blood pressure of the participants was measured with an automated oscillometric upper-arm blood-pressure monitor (Omron 705IT, Omron Corporation, Japan). To ensure accurate measurements, cuffs adapted for children were used depending on the child’s right upper arm circumference. Measurements were performed by a standardized method, as described by the European Society of Hypertension [27]. In summary, after the child had rested for 10 min in supine position, a trained observer obtained five consecutive readings of the systolic (SBP) and diastolic (DBP) blood pressure of the right arm, with 1-min intervals. Average SBP and DBP were based on the last three readings. Mean arterial pressure (MAP) was calculated via the equation: MAP = (2/3 × DBP) + (1/3 × SBP).

We used retinal vascular imaging as a proxy to examine the systemic microvasculature between the age of four and six. This approach has been proven successful in other studies that examined early lifestyle factors related to cardiovascular disease [28]. To determine the retinal blood vessel parameters, fundus pictures of the oculus dextrus and oculus sinister were taken with a Canon CR-2 plus 45° 6.3 megapixel digital non-mydriatic retinal camera (Hospithera, Brussels, Belgium). These pictures were subsequently analyzed with the MONA® software (version 2.1.1, VITO Health, Mol, Belgium). First, the diameter of the optic disk (OD) was determined for each picture, since all distance measurements within the fundus were set relative to this value. Next, the widths of the retinal arterioles and venules were calculated within the predetermined area of 0.5 and 1 times the OD diameter, starting from the margin of the optic disk (Additional file 1: Fig. S2). The diameters of the six largest arterioles and six largest venules in this zone were used in the revised Parr-Hubbard formula to calculate the central retinal arteriolar equivalent (CRAE) and central retinal venular equivalent (CRVE) [29]. The tortuosity index (TI) of the retinal vasculature was determined between 1.5 and 5 times the radius of the OD and can be described as a measure for the curvature of the retinal vessels in this zone. The normalized tortuosity is calculated as the average tortuosity of the branch segments, where the tortuosity of a branch segment is the ratio of the line traced on each tree along the vessel axis between 0.5 and 2 times the OD diameter and the line connecting the endpoints. Segmentations are cropped centered on the OD whereby the inner and outer radii were taken at 1.5 and 5.0 times the radius of the OD [30].

Of the 332 mother-child pairs, 74 were not included in the statistical analyses since no (good quality) images of the retinal vasculature of either one eye were available (Additional file 1: Fig. S1). Pictures could not be taken due to the participant having autism spectrum disorder (*n* = 2), severe mental retardation (*n* = 1), blindness (*n* = 1), and lack of concentration or unwillingness to participate (*n* = 20). For 50 participants, the quality of the pictures was suboptimal, due to children’s movement of their body or eyes during the capturing of the images. Of the remaining 258 mother-child pairs, the data on alcohol use during pregnancy were missing for three mothers, and for ten children, no blood pressure could be determined at the moment of the follow-up visit. Therefore, these 13 participants were excluded, and final statistics were performed on 245 mother-child pairs.

### Exposure assessment

The maternal residential address was used to interpolate regional background levels of PM_2.5_ and NO_2_ concentrations (μg/m^3^) during pregnancy and for the postnatal period, based on a high-resolution spatial-temporal interpolation method [31], as described in the cohort profile paper of Janssen et al. The overall model performance was assessed by leave-one-out cross-validation, including 44 monitoring stations for NO_2_ and 34 stations for PM_2.5_. The validation statistics of the interpolation tool explained more than 78% of the temporal and spatial variability in Flanders for NO_2_ and 80% for PM_2.5_ [32, 33]. Furthermore, accuracy of our exposure models was recently proven by the correlation between the urinary load of nano-sized black carbon particles in children and residential levels of PM_2.5_ and NO_2_ [34], and for prenatal exposure by a correlation between this exposure model and the placental black carbon load [4].

The described model provided daily air pollution levels for each participant. Daily values were averaged for each specific time window during the pregnancy: first trimester (i.e., date of conception until 13 weeks of pregnancy), second trimester (i.e., 14 weeks until 26 weeks of pregnancy), third trimester (i.e., 27 weeks of pregnancy until delivery), and the entire period of pregnancy, from the date of conception until the date of delivery. Ultrasound imaging data combined with the first day of the mother’s last menstrual period were used to estimate the date of conception [35]. Postnatal PM_2.5_ and NO_2_ exposures were averaged for the day of the follow-up visit, the day before the follow-up visit, and the week before the follow-up visit as short-term exposure windows, and the average exposure during childhood (i.e., the average daily exposure from the day of birth until the day before the follow-up visit) as long-term exposure window. Exposure data during childhood were available for 226 out of the 245 participants. For mothers who moved during pregnancy (*n* = 21) and between birth and follow-up examination (*n* = 69), we calculated the specific exposures, allowing for the changes in the corresponding period.

### Statistics

We used the SAS software (version 9.4; SAS Institute Inc., Cary, NC, USA) for data analysis. Normality and equality of variances, as assumptions of model linearity, were tested with the Shapiro-Wilk statistic and Q-Q plots of the residuals, respectively. Differences in characteristics between participants and non-participants of the follow-up study were tested by means of a two-sided *t* test for continuous variables and with a chi-square test for the categorical variables. Unadjusted correlations between prenatal exposure to PM_2.5_ and NO_2_, and the retinal microvascular characteristics, as well as between prenatal and postnatal air pollution levels, were examined by means of Pearson’s correlation. In the main analyses, the association of retinal vessel widths (CRAE and CRVE) and vessel curvature (TI) with either prenatal or postnatal PM_2.5_ and NO_2_ exposures was assessed using multivariable linear regression modeling. Pregnancy trimester-averaged PM_2.5_ and NO_2_ exposure levels were entered into the same model in order to estimate independent trimester-specific effects. Minimally adjusted models were adjusted for sex and age (years), and we adjusted the analyses for the following variables: age (years), sex, ethnicity, mean arterial blood pressure and BMI of the child at the moment of the follow-up visit, the season in which the follow-up examination took place, birth weight (grams), the age of the mother during pregnancy and her pre-pregnancy BMI, the education level of the mother, alcohol use of the mother during pregnancy, the smoking habits of the mother before and during pregnancy, and the exposure of the child to passive smoking. The interaction between whole pregnancy air pollution exposure and age, sex, MAP, and BMI on the microvascular parameters was explored using continuous variables. We found a significant interaction for MAP and prenatal NO_2_ exposure on CRAE and CRVE; therefore, we additionally constructed a variable indicating high and low MAP (based on the median) and stratified the analysis accordingly. In the secondary analyses, we combined entire pregnancy exposure and postnatal exposures during either the day of the follow-up examination, the week before the follow-up visit, or the entire childhood exposure in the same model. The magnitude of all associations was expressed for an interquartile range (IQR; between the 25th and 75th percentile) increase in the observed exposure.

We performed three separate sensitivity analyses. Firstly, a sensitivity analysis was conducted to assess the association between prenatal air pollution exposure and the CRVE, CRAE, and TI in a population excluding mothers with diagnosed hypertension during pregnancy (*n* = 8) and those with gestational diabetes (*n* = 15). Furthermore, Wei and colleagues found that prematurity could also affect the retinal vessel characteristics of children later in life [36]. Therefore, we performed an additional sensitivity analysis excluding the children who were born before 37 weeks of gestation (*n* = 12). Finally, a third sensitivity analysis was conducted, excluding mothers who smoked during pregnancy (*n* = 32).

## Results

### Study population characteristics

Background characteristics of the 242 non-participants were similar to those of the participants with analyzed retinal images and full data (*n* = 245) and the participants of the follow-up study with poor quality pictures or without full data (*n* = 87) with respect to parity, pre-pregnancy BMI and smoking behavior, child’s sex distribution, birth weight, and birth length (Additional file 1: Table S1). However, mothers that renewed consent were significantly older at the birth of their child and were more likely to have a higher education level and to be of European ancestry compared with non-participants. Prenatal levels of residential particulate air pollution by trimester did not differ, while the total gestational exposure was on average slightly higher in participants compared with non-participants. For prenatal NO_2_, none of the exposure windows showed difference between participants and non-participants. The postnatal PM_2.5_ and NO_2_ exposures did not differ between the three groups.

The characteristics of the 245 mother-child pairs participating in this study are summarized in Table 1. At the time of birth, the mothers had an average (standard deviation) age of 29.9 (4.1) years. Their pre-pregnancy BMI was 24.4 (4.6) kg/m^2^. Most of the mothers had never smoked (*n* = 167; 68.2%) or stopped smoking before pregnancy (*n* = 46; 18.8%), and 78.8% of the mothers did not consume alcohol throughout their pregnancy. Almost three quarters of all women were highly educated (67.4%), having a college degree or higher.

**Table 1:** Average (SD) or numbers (%) of the characteristics of the mother-child pairs included in this study (*n* = 245), and the girls (*n* = 129) and boys (*n* = 116) separately. The *p* value depicts the difference between girls and boys

Characteristic	Combined	Girls	Boys	*p* value
Mother				
Age at birth child, years	29.9 (4.1)	30.1 (4.1)	29.7 (4.1)	0.41
Pre-pregnancy BMI, kg/m^2^	24.4 (4.6)	24.4 (4.9)	24.3 (4.3)	0.78
Smoking behavior during pregnancy				0.48
Never smoked	167 (68.2)	90 (36.8)	77 (31.4)	
Stopped smoking before pregnancy	46 (18.8)	24 (9.8)	22 (9.0)	
Smoked during pregnancy	32 (13.0)	15 (6.1)	17 (6.9)	
Alcohol consumption during pregnancy				0.67
Yes	52 (21.2)	26 (10.6)	26 (10.6)	
No	193 (78.8)	103 (42.1)	90 (36.7)	
Education level				0.59
Low (no high school diploma)	16 (6.5)	7 (2.9)	9 (3.7)	
Middle (high school diploma)	64 (26.1)	34 (13.9)	30 (12.2)	
High (college degree or higher)	165 (67.4)	88 (35.9)	77 (31.4)	
Child				
Ethnicity				0.26
European	230 (93.9)	119 (48.6)	111 (45.3)	
Non-European	15 (6.1)	10 (4.1)	5 (2.0)	
Birth weight, g	3446.6 (429.8)	3413.3 (412.9)	3483.5 (446.8)	0.20
Age at time of follow-up, years	4.6 (0.4)	4.6 (0.4)	4.5 (0.4)	0.75
Height at time of follow-up, cm	107.3 (4.9)	107.9 (5.1)	107.8 (4.6)	0.81
Weight at time of follow-up, kg	18.7 (2.7)	18.8 (2.7)	18.7 (2.7)	0.70
BMI at time of follow-up, kg/m^2^	16.0 (1.4)	16.1 (1.4)	16.0 (1.4)	0.68
Season at follow-up				0.82
Spring	83 (33.9)	46 (18.8)	37 (15.1)	
Summer	48 (19.6)	25 (10.2)	23 (9.4)	
Autumn	42 (17.1)	18 (7.3)	24 (9.8)	
Winter	72 (29.4)	40 (16.3)	32 (13.1)	
Systolic blood pressure, mmHg	97.6 (8.2)	98.1 (8.0)	97.0 (8.5)	0.30
Diastolic blood pressure, mmHg	53.9 (6.9)	54.5 (6.6)	53.2 (7.1)	0.13
Mean arterial pressure, mmHg	68.5 (6.0)	69.1 (5.6)	67.8 (6.3)	0.10
Exposure to passive smoking				0.43
Not exposed	170 (69.4)	88 (35.9)	82 (33.5)	
Exposed via one parent	46 (18.8)	23 (9.4)	23 (9.4)	
Exposed via both parents	29 (11.8)	18 (7.3)	11 (4.5)	
CRAE, μm	180.8 (14.2)	182.5 (13.0)	178.9 (15.3)	0.05
CRVE, μm	251.0 (19.4)	253.7 (18.1)	247.9 (20.5)	0.02
TI	0.889 (0.012)	0.891 (0.012)	0.887 (0.013)	0.04

*Abbreviations*: *CRAE* central retinal arteriolar equivalent, *CRVE* central retinal venular equivalent, *TI* tortuosity index, *SD* standard deviation

At the moment of the follow-up examination, the children (52.6% girls) had an average age of 4.6 (0.4) years, an average height of 107.3 (4.9) cm, and an average weight of 18.7 (2.7) kg. Mean arterial pressure averaged 68.5 (6.0) mmHg. Most of the follow-up visits took place during spring (33.9%) and winter (29.4%). Most children were not exposed to passive smoking in their home environment (*n* = 170), while 18.8% of the children were exposed via one parent and 11.8% of the children were exposed to passive smoking via both parents.

### Exposure characteristics

Table 2 summarizes the PM_2.5_ and NO_2_ exposure characteristics of the study population during pregnancy and early childhood. The average exposures for both air pollution components were comparable between the three trimesters and the entire pregnancy. For PM_2.5_, an average exposure (IQR) of 14.3 (12.6–15.8) μg/m^3^ was calculated for the entire pregnancy, and for the same period, the average NO_2_ level was 19.7 (16.5–22.7) μg/m^3^. The average (IQR) childhood exposure level calculated for PM_2.5_ was 12.6 (11.9–13.3) μg/m^3^, while for NO_2_, this was 17.2 (14.6–19.3) μg/m^3^. All PM_2.5_ and NO_2_ exposures of the three trimesters and whole pregnancy were strongly correlated (*p* ≤ 0.02), except for the first and third trimester NO_2_ (*r* = − 0.07, *p* = 0.29), third trimester NO_2_ and second trimester PM_2.5_ (*r* = 0.03, *p* = 0.65), and first trimester PM_2.5_ and whole pregnancy NO_2_ exposure (*r* = − 0.04, *p* = 0.51). A strong correlation was identified for exposure during the entire pregnancy and lifetime of the children, both for PM_2.5_ (*r* = 0.32, *p* < 0.0001) and NO_2_ exposures (*r* = 0.81, *p* < 0.0001).

**Table 2:** Exposure details on PM_2.5_ and NO_2_ air pollution (μg/m^3^) during different time windows of pregnancy and different periods during the childhood of the participants (*n* = 245)

Type of air pollution exposure and time window	Mean (SD)	25th percentile	75th percentile	IQR
PM_2.5_				
Pregnancy				
Trimester 1	14.3 (5.5)	9.6	18.2	8.6
Trimester 2	14.2 (5.1)	9.6	17.9	8.3
Trimester 3	14.2 (5.7)	9.1	18.3	9.2
Entire pregnancy	14.3 (2.3)	12.6	15.8	3.2
Childhood				
Day of follow-up visit	11.2 (8.3)	4.8	15.1	10.3
Day before follow-up visit	12.1 (10.2)	4.5	15.9	11.4
Week before follow-up visit	13.0 (7.5)	7.3	17.7	10.4
Average childhood exposure	12.6 (1.1)	11.9	13.3	1.4
NO_2_				
Pregnancy				
Trimester 1	19.9 (6.0)	15.2	24.3	9.1
Trimester 2	19.8 (6.2)	15.0	24.1	9.1
Trimester 3	19.6 (6.2)	14.7	23.7	9.0
Entire pregnancy	19.7 (4.4)	16.5	22.7	6.2
Childhood				
Day of follow-up visit	17.0 (8.7)	10.5	21.3	10.8
Day before follow-up visit	17.2 (9.5)	9.7	22.8	13.1
Week before follow-up visit	17.2 (7.2)	11.7	21.7	10.0
Average childhood exposure	17.2 (3.4)	14.6	19.3	4.7

*Abbreviations*: *IQR* interquartile range, *NO*_*2*_ nitrogen dioxide, *PM*_*2*.5_ particulate matter with a diameter smaller than 2.5 μm, *SD* standard deviation

### Microvasculature characteristics

The average CRAE, CRVE, and TI values of both eyes were used for each child if both pictures were available. For 200 participants, the retina pictures of both eyes were used; for 27 individuals, only that of the left eye; and for 18 children, only the picture of the right eye was available for analysis since the picture of the other eye was of insufficient quality. There was no difference between the values of either one or two eyes for the CRAE (*p* = 0.38), CRVE (*p* = 0.38), or TI (*p* = 0.38).

For all children, the average (SD) CRAE and CRVE were 180.8 (14.2) μm and 251.0 (19.4) μm, respectively, and the average TI was 0.889 (0.012) (Table 1). The CRAE, CRVE, and TI were slightly higher in girls than in boys (Table 1). A positive correlation was found between both CRVE and CRAE (*r* = 0.60, *p* < 0.0001), between CRVE and TI (*r* = 0.19, *p* = 0.003), and between CRAE and TI (*r* = 0.14, *p* = 0.02).

### Main analyses

#### Associations between prenatal air pollution exposure and retinal microvasculature

**Fig. 1:**
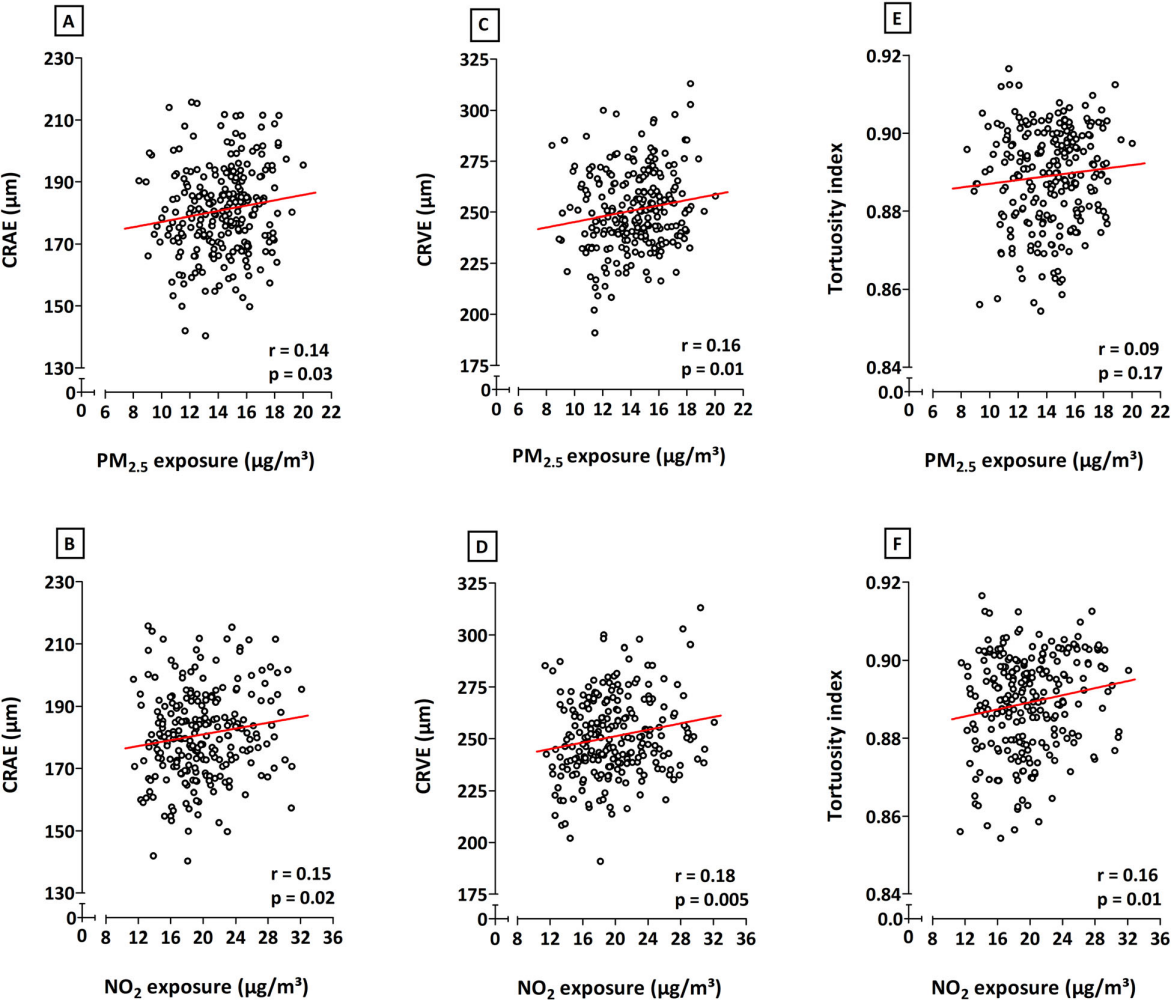
Correlations between exposure to either PM_2.5_ (first row) or NO_2_ (second row) during the entire pregnancy and CRAE (**a**, **b**), CRVE (**c**, **d**), and tortuosity index (**e**, **f**). The respective *r* and *p* values are depicted on each plot. *Abbreviations*: CRAE, central retinal arteriolar equivalent; CRVE, central retinal venular equivalent; NO_2_, nitrogen dioxide; PM_2.5_, particulate matter with a diameter smaller than 2.5 μm

Correlations analyses, without additional adjustments, showed positive relationships between in utero exposure to both PM_2.5_ and NO_2_ during the entire pregnancy and the CRAE, CRVE, and TI (Fig. 1). Only the association between entire pregnancy exposure to PM_2.5_ and the tortuosity of the retinal vessels was not significant (*p* ≤ 0.05). Correlations between microvascular traits and PM_2.5_ or NO_2_ exposure during the separate trimesters of pregnancy were only significant between the third trimester NO_2_ exposure and TI (data not shown).

**Fig. 2:**
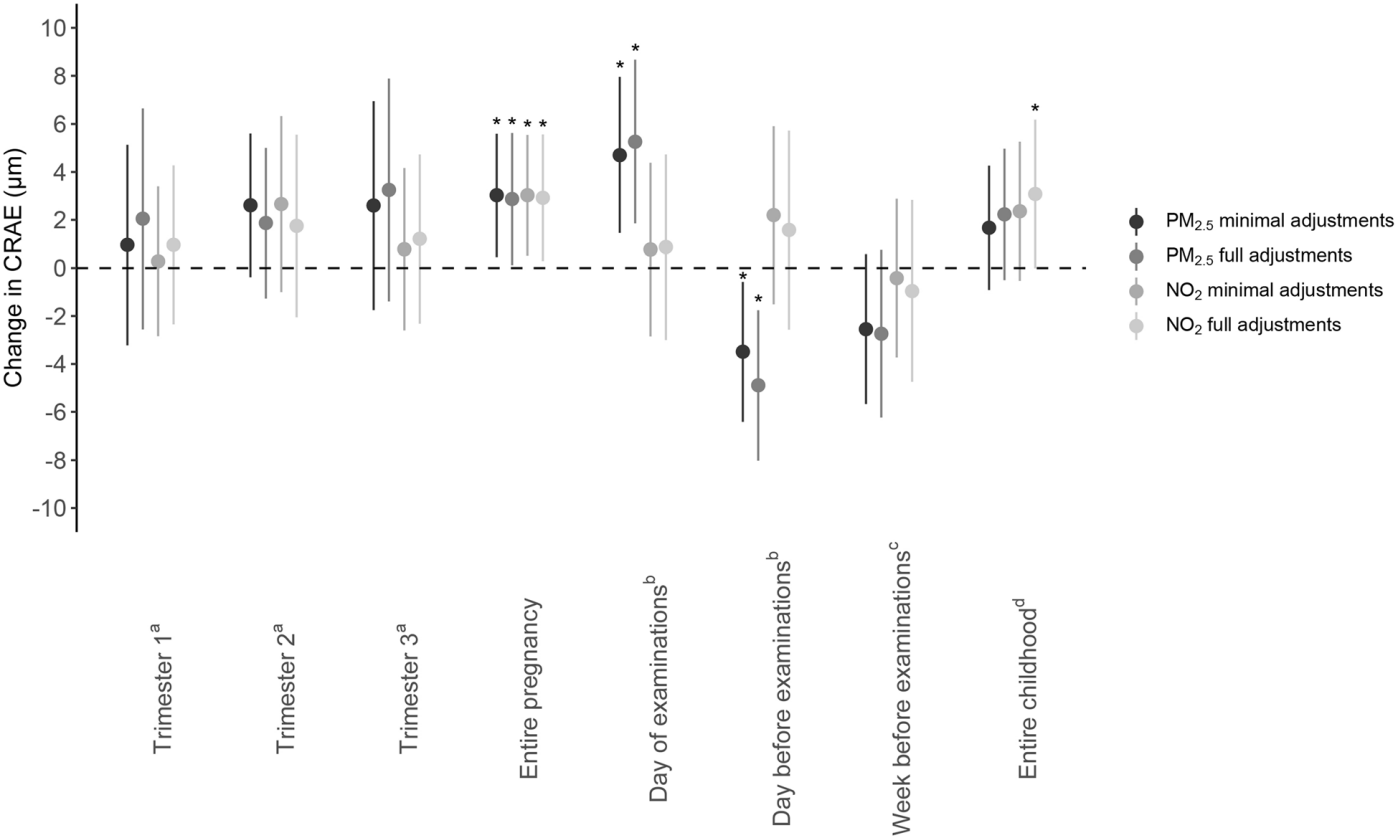
Associations between CRAE and PM_2.5_ or NO_2_ exposure during pregnancy or during childhood. Estimates are given as change (95% CI) for every IQR increase in PM_2.5_ (two darker gray dots) or NO_2_ (two lighter gray dots). Minimally adjusted models were adjusted for sex and age (years); fully adjusted models were adjusted for age (years), sex, ethnicity, mean arterial blood pressure and BMI of the child at the moment of the follow-up visit, the season in which the follow-up examination took place, birth weight (grams), maternal age at the birth of her child and pre-pregnancy BMI, maternal education level, alcohol use of the mother during pregnancy, smoking habits of the mother before and during pregnancy, and the exposure of the child to passive smoking. ^a^Model adjusted for the three pregnancy trimester-averaged exposures levels. ^b^Model adjusted for exposure on the day of the follow-up visit and exposure of the day preceding the follow-up visit. ^c^Model adjusted for exposure on the day of the follow-up visit and exposure of the week preceding the follow-up visit. ^d^Model adjusted for exposure on the day of the follow-up visit and average childhood exposure from the day of birth until the day before the follow-up examination. *Abbreviations*: CI, confidence interval; CRAE, central retinal arteriolar equivalent; IQR, interquartile range; NO_2_, nitrogen dioxide; PM_2.5_, particulate matter with a diameter smaller than 2.5 μm. *p ≤ 0.05

**Fig. 3:**
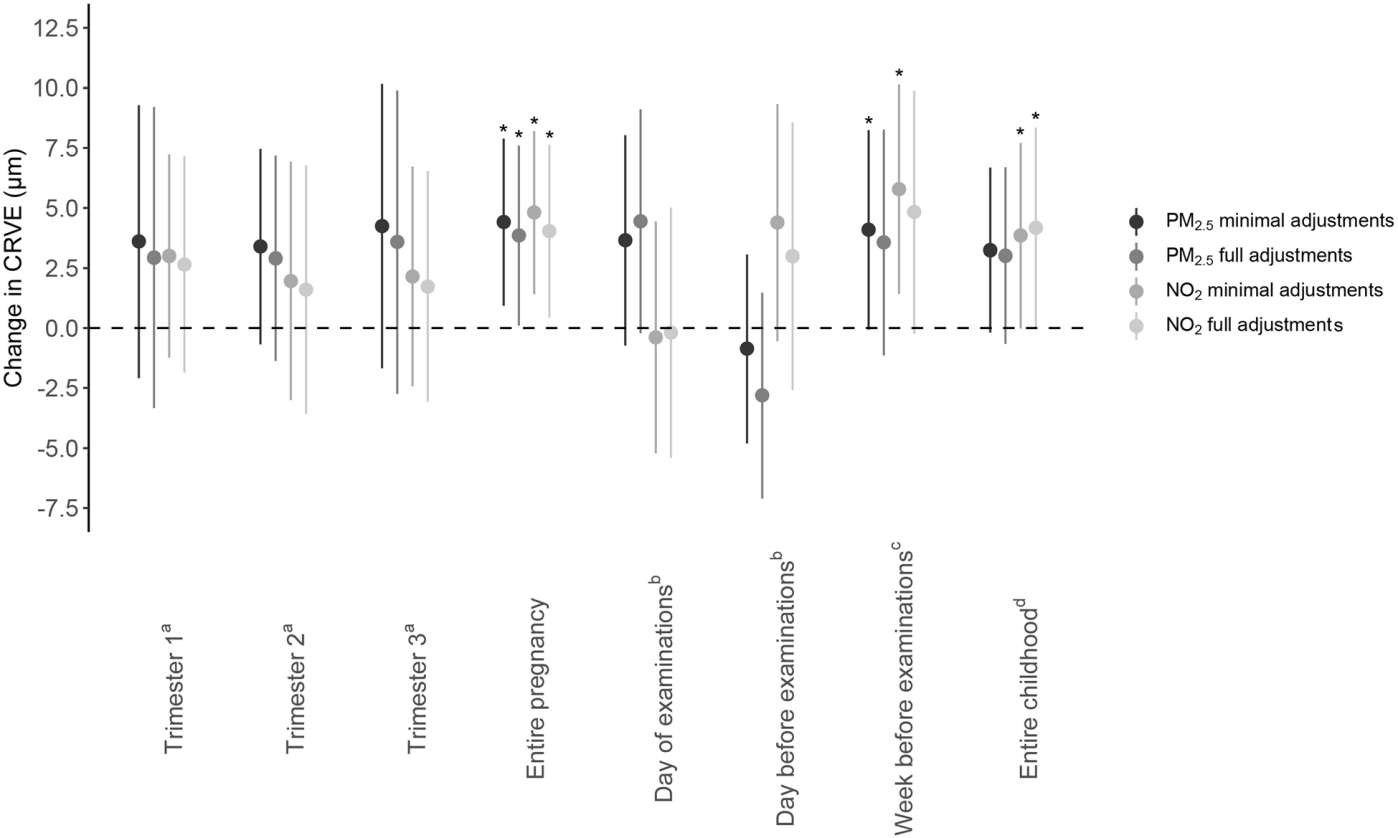
Associations between CRVE and PM_2.5_ or NO_2_ exposure during pregnancy or during childhood. Estimates are given as change (95% CI) for every IQR increase in PM_2.5_ (two darker gray dots) or NO_2_ (two lighter gray dots). Minimally adjusted models were adjusted for sex and age (years); fully adjusted models were adjusted for age (years), sex, ethnicity, mean arterial blood pressure and BMI of the child at the moment of the follow-up visit, the season in which the follow-up examination took place, birth weight (grams), maternal age at the birth of her child and pre-pregnancy BMI, maternal education level, alcohol use of the mother during pregnancy, smoking habits of the mother before and during pregnancy, and the exposure of the child to passive smoking. ^a^Model adjusted for the three pregnancy trimester-averaged exposures levels. ^b^Model adjusted for exposure on the day of the follow-up visit and exposure of the day preceding the follow-up visit. ^c^Model adjusted for exposure on the day of the follow-up visit and exposure of the week preceding the follow-up visit. ^d^Model adjusted for exposure on the day of the follow-up visit and average childhood exposure from the day of birth until the day before the follow-up examination. *Abbreviations*: CI, confidence interval; CRVE, central retinal venular equivalent; IQR, interquartile range; NO_2_, nitrogen dioxide; PM_2.5_, particulate matter with a diameter smaller than 2.5 μm. *p ≤ 0.05

**Fig. 4:**
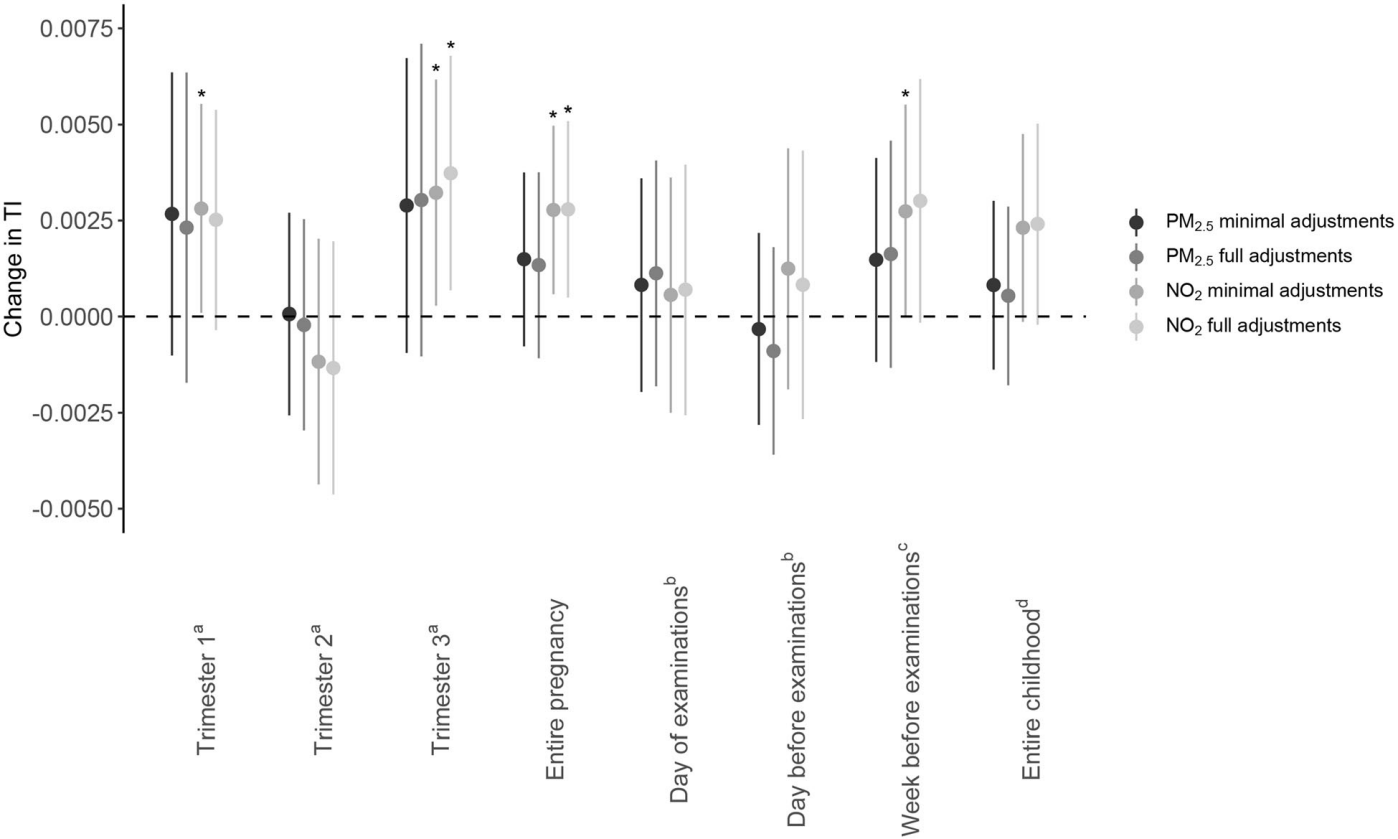
Associations between TI and PM_2.5_ or NO_2_ exposure during pregnancy or during childhood. Estimates are given as change (95% CI) for every IQR increase in PM_2.5_ (two darker gray dots) or NO_2_ (two lighter gray dots). Minimally adjusted models were adjusted for sex and age (years); fully adjusted models were adjusted for age (years), sex, ethnicity, mean arterial blood pressure and BMI of the child at the moment of the follow-up visit, the season in which the follow-up examination took place, birth weight (grams), maternal age at the birth of her child and pre-pregnancy BMI, maternal education level, alcohol use of the mother during pregnancy, smoking habits of the mother before and during pregnancy, and the exposure of the child to passive smoking. ^a^Model adjusted for the three pregnancy trimester-averaged exposures levels. ^b^Model adjusted for exposure on the day of the follow-up visit and exposure of the day preceding the follow-up visit. ^c^Model adjusted for exposure on the day of the follow-up visit and exposure of the week preceding the follow-up visit. ^d^Model adjusted for exposure on the day of the follow-up visit and average childhood exposure from the day of birth until the day before the follow-up examination. *Abbreviations*: CI, confidence interval; IQR, interquartile range; NO_2_, nitrogen dioxide; PM_2.5_, particulate matter with a diameter smaller than 2.5 μm; TI, tortuosity index. *p ≤ 0.05

Multiple linear regression modeling showed a positive association between entire pregnancy exposure to PM_2.5_ and CRAE in both minimally and fully adjusted models (Fig. 2). With full adjustments, for every IQR increase in PM_2.5_ air pollution exposure during pregnancy, a 2.87-μm widening of the arteriolar diameter was observed (95% CI, 0.12 to 5.62; *p* = 0.04). For whole pregnancy exposure to NO_2_, a widening of 2.92 μm of the arterial diameter was determined for every IQR increase in exposure (95% CI, 0.29 to 5.56; *p* = 0.03). No significant changes in CRAE were observed for increased levels of either NO_2_ or PM_2.5_ exposure during the three separate trimesters of pregnancy. An IQR increment in prenatal air pollution exposure during the entire pregnancy was associated with a 3.85-μm (95% CI, 0.10 to 7.60; *p* = 0.04) higher CRVE for PM_2.5_ and a 4.03-μm (95% CI, 0.44 to 7.63; *p* = 0.03) widening of the retinal venules for NO_2_ exposure in the fully adjusted models (Fig. 3). Again, as observed for the CRAE, no significant changes in CRVE were determined in association with trimester-specific exposure. PM_2.5_ exposure during pregnancy was not associated with the tortuosity index. However, an association between TI and in utero NO_2_ exposure was found in both minimally and fully adjusted models. In fully adjusted models, an IQR increase in prenatal NO_2_ exposure over the entire pregnancy was associated with a 0.0028 (95% CI, 0.0005 to 0.0051, *p* = 0.02) higher TI, which was mainly driven by the exposure in the third trimester (Fig. 4).

With same full adjustments as before, we explored the interaction between age, sex, MAP and BMI, and microvascular parameters using continuous variables. These interaction terms were all non-significant (*p* ≥ 0.18) except borderline significance for mean arterial pressure in association with both CRAE and CRVE for whole pregnancy NO_2_ exposure. Categorizing MAP above and below the median revealed stronger prenatal NO_2_ exposure effects on CRAE and CRVE associations in children in the high MAP group (Additional file 1: Table S2).

#### Associations between postnatal PM_2.5_ and NO_2_ exposures and retinal microvasculature

Exposure to PM_2.5_ on the day of the follow-up examination and the day before the visit had a significant effect on the CRAE (Fig. 2) but not on the CRVE (*p* ≥ 0.10) (Fig. 3). The arterial diameter was 5.26 μm wider (95% CI, 1.86 to 8.67, *p* = 0.003) for every IQR increase in PM_2.5_ exposure on the day of the measurements, while a narrowing of 4.89 μm (95% CI, − 8.02 to − 1.76, *p* = 0.002) was determined for every IQR higher PM_2.5_ exposure on the day before the follow-up visit. No associations were found between NO_2_ exposure and either CRAE, CRVE, or TI for exposure on the day on which, or the day before, the retinal images were taken. When the exposure of the week before the follow-up examination was considered, an association between NO_2_ exposure and CRVE could be identified in the minimally adjusted model, which disappeared following full adjustment (Fig. 3). In case of average exposure during the lifetime of the children, in fully adjusted models, we observed a 3.08-μm (95% CI, − 0.01 to 6.18, *p* = 0.05) widening of the CRAE (Fig. 2) and a 4.17-μm (95% CI, − 0.01 to 8.35, *p* = 0.05) widening of the CRVE (Fig. 3) for every IQR increase in NO_2_ during the child’s lifetime.

### Secondary analyses

#### Associations between retinal microvasculature and residential air pollution in combined prenatal and postnatal exposure models

In the secondary analyses, we combined prenatal exposure during the entire pregnancy and postnatal exposure to either PM_2.5_ or NO_2_ in one model. NO_2_ exposure during the entire gestation was correlated with both short-term exposure (day of the follow-up, day before the follow-up, and week before follow-up; *r* = 0.35, *r* = 0.34, and *r* = 0.45 respectively, *p* < 0.0001) and long-term exposure (entire childhood; *r* = 0.81, *p* < 0.0001) to ambient NO_2_. For PM_2.5_, we only noted a correlation between entire pregnancy exposure and postnatal exposure during the entire childhood (*r* = 0.32, *p* < 0.0001).

Only the associations between postnatal exposure to PM_2.5_ on the day of, or the day before, the follow-up visit and CRAE remained significant (*p* = 0.003) in models mutually adjusted for exposure during the entire pregnancy (results not shown). However, adjusting modeled entire pregnancy exposure for short-term postnatal exposure (i.e., on the day of the follow-up, or on the day or the week before the examination) increased the effect estimates for both the CRAE and CRVE, while the estimates for the TI did not substantially change. Moreover, the associations with entire pregnancy exposure and the retinal vessel diameter and tortuosity remained, except for CRVE and TI after adjustment of the model for NO_2_ exposure during the day or in the week before the follow-up examination (Table 3). Including exposure during the entire childhood into the model decreased the estimates of both the CRVE and TI in association with every IQR increase in whole pregnancy PM_2.5_ and NO_2_ exposures and that of the CRAE in association with prenatal NO_2_, while a higher estimate for the CRAE was found in association with increased prenatal PM_2.5_ exposure after mutual adjustment for entire childhood PM_2.5_ exposure. Only the latter association remained significant in the model including whole postnatal air pollution exposure (Table 3).

**Table 3:** Associations between retinal microvascular characteristics and PM_2.5_ or NO_2_ exposure during pregnancy: results from secondary analyses with models combining entire pregnancy exposure with different postnatal exposure periods

Model	CRAE, μm	CRVE, μm	TI
PM_2.5_			
Entire pregnancy + day of follow-up	3.76 (0.76 to 6.75)*	4.29 (0.24 to 8.33)*	0.0022 (− 0.0003 to 0.0047)
Entire pregnancy + day before follow-up	3.75 (0.75 to 6.74)*	4.32 (0.26 to 8.39)*	0.0022 (− 0.0003 to 0.0048)
Entire pregnancy + week before follow-up	3.73 (0.71 to 6.74)*	4.71 (0.68 to 8.74)*	0.0024 (− 0.0002 to 0.0049)
Entire pregnancy + entire childhood	3.25 (0.13 to 6.37)*	3.53 (− 0.69 to 7.74)	0.0022 (− 0.0005 to 0.0048)
NO_2_			
Entire pregnancy + day of follow-up	2.96 (0.05 to 5.87)*	4.24 (0.32 to 8.16)*	0.0027 (0.0002 to 0.0051)*
Entire pregnancy + day before follow-up	2.89 (− 0.01 to 5.79)*	3.90 (− 0.01 to 7.81)*	0.0027 (0.0002 to 0.0051)*
Entire pregnancy + week before follow-up	3.83 (0.74 to 6.91)*	3.25 (− 0.90 to 7.40)	0.0021 (− 0.0005 to 0.0047)
Entire pregnancy + entire childhood	1.68 (− 3.22 to 6.58)	3.09 (− 3.51 to 9.70)	0.0024 (− 0.0017 to 0.0065)

Estimates are given as change (95% CI) per IQR increase in exposure to either PM_2.5_ or NO_2_ during the entire pregnancy. All models were adjusted for exposure to either PM_2.5_ or NO_2_ during the entire pregnancy, age (years), sex, ethnicity, mean arterial blood pressure and BMI of the child at the moment of the follow-up visit, the season in which the follow-up examination took place, birth weight (grams), maternal age at the birth of her child and pre-pregnancy BMI, maternal education level, alcohol use of the mother during pregnancy, smoking habits of the mother before and during pregnancy, and the exposure of the child to passive smoking. The separate models were additionally adjusted for exposure during either the day of the follow-up visit, the day before the follow-up examination, the week preceding the follow-up visit, or the average childhood exposure from the day of birth until the day of the follow-up examination. *Abbreviations*: *CI* confidence interval, *CRAE* central retinal arteriolar equivalent, *CRVE* central retinal venular equivalent, *TI* tortuosity index

*p ≤ 0.05

### Sensitivity analyses

Excluding mothers who were diagnosed with hypertension (*n* = 8) and gestational diabetes (*n* = 15) during pregnancy slightly decreased the reported estimates of all three retinal vessel characteristics, in association with both of the examined whole pregnancy exposures (Additional file 1: Table S3). The associations between the entire pregnancy PM_2.5_ exposure and both CRVE and CRAE, and between NO_2_ exposure during the entire gestation and CRAE lost their significance (*p* > 0.06). Similar findings could be concluded for the sensitivity analysis excluding newborns with a gestational age lower than 37 weeks (*n* = 12) (Additional file 1: Table S4). However, the association between entire pregnancy NO_2_ exposure and CRAE remained significant in this subgroup. Finally, a third sensitivity analysis excluding mothers who had smoked during pregnancy (*n* = 30) did not substantially alter the described relationships between the retinal vessel characteristics and either PM_2.5_ or NO_2_ exposure during pregnancy (Additional file 1: Table S5).

## Discussion

We have evaluated the associations between gestational and childhood exposures to ambient air pollution and microvascular structure by using retinal vessel metrics in 4- to 6-year-old children. The key findings are as follows: (1) retinal venular and arterial diameters of children widen with a higher exposure of their mother to PM_2.5_ and NO_2_ during the entire pregnancy period, and (2) the retinal blood vessel curvature is affected by in utero exposure to NO_2_, represented by an increase in tortuosity index for the entire pregnancy and the third trimester. To our knowledge, we are the first to find an association between air pollution exposure during gestation and effects on the retinal microvasculature later in life.

Several studies found associations between retinal vascular characteristics and both acute and chronic exposure to air pollution in middle-aged or older populations. One of these studies was performed in a population of healthy adults with an average age of 64 from the Multi-Ethnic Study of Atherosclerosis (MESA) cohort study [21]. In this group, narrower retinal arterioles and wider venules were observed with increased 2-year exposure to PM_2.5_. A Belgian study focused on the effects of short-term air pollution exposure on retinal microcirculation in adults between the age of 22 and 63. Both the average CRAE and CRVE in this research decreased with increasing exposure to PM_10_ and black carbon [37]. These results were further supported and even associated with changes in specific miRNAs linked to inflammatory and oxidative stress pathways [38]. Provost et al. were the first to describe the relationship between retinal vessel diameter and both short-term and long-term exposures to PM_2.5_ air pollution in children [25]. They determined exposures on the day of the retinal examinations, as well as in the year prior to their measurements. In accordance with our results, the authors found that there was a more significant effect of short-term exposure on the CRAE, although they found an association with a narrowing of the retinal arterioles, in contrast to our population. For long-term average annual exposure, no effects could be observed on both the CRAE and CRVE in this population in 8- to 12-year-old children.

Although studies focus on different forms and time windows of ambient air pollution exposure during the lifetime, there is a clear indication that the diameter of retinal venules is affected. Several systemic and environmental factors have been attributed to a wider CRVE over the course of life. For example, retinal venular widening has been associated not only with the effects of active smoking but also with systemic diseases such as diabetes and obesity [39, 40]. Research conducted within the Rotterdam Study, in a population of people over the age of 55, showed that both the venular and arteriolar retinal diameters widened when the participants had formerly smoked or were active smokers, with the largest effect on the retinal venules [41]. Widening of the retinal venules has also been considered as a potential biomarker for adverse health conditions. A meta-analysis combining the results of six individual prospective cohort studies has shown that a wider CRVE can be an indicator of coronary heart disease in adult women [42]. Furthermore, a recent long-term follow-up cohort study has described that the width of retinal venules could be a potential predictor of ischemic stroke in both men and women and, in accordance with the former meta-analysis, of coronary heart disease in women [18].

In this research, we have described a positive association between CRAE and prenatal exposure to PM_2.5_ and NO_2_. In the context of exposure to air pollution, studies on changes in CRAE mostly seem to show negative relationships between the exposure variable and retinal arterial diameter [21, 37, 38]. Indeed, a narrower CRAE has been associated with several detrimental cardiovascular health outcomes, such as hypertension and arterial stiffness [43]. However, environmental exposures or adverse conditions associated with an increase of the CRAE have also been described over the past years. A wider CRAE was linked with high cholesterol in a population of the Locomotive Syndrome and Health Outcome in Aizu Cohort Study [40] and with cigarette smoking in the Multi-Ethnic Study of Atherosclerosis (MESA) study. Potential mechanisms explaining these effects are endothelial dysfunction and elevated oxidative stress as observed in mouse models [44]. A widening of the retinal arterial diameter has also been associated with several disease outcomes. Rhee et al. found that people who were diagnosed with intracranial arterial stenosis had a higher CRAE compared to those without the condition [45], while another study conducted within an Asian population showed an association between wider CRAE values and a higher incidence of diabetes [46]. Since both narrowing and widening of the retinal arterioles have been associated with detrimental health outcomes later in life, our findings should be traced further within the follow-up cohort, to be able to evaluate the changes in CRAE and the correlated health changes in these children throughout their life course.

Not only the diameter of the retinal vessels was described to be affected by exposure to air pollution in utero. In this study, the tortuosity of the vessel network was found to increase with higher exposure to NO_2_ during the entire period of pregnancy. Tortuosity can be regarded as a measure for vessel curvature and has been found to be influenced by conditions such as diabetes and hypertension [47]. Although this microvascular characteristic has been studied to a lesser extent than the retinal vascular diameter, with an apparent lack of studies on the relation to environmental exposures, vessel tortuosity has also been identified as a potential marker for the risk of developing cardiovascular disease. For example, higher microvascular tortuosity in the retina has been associated with an increased risk of developing cerebral microbleeds [48] and ischemic stroke [49].

The World Health Organization (WHO) and European limits that have been determined on the short-term (1-h mean) and long-term (annual mean) exposures to NO_2_ are 200 and 40 μg/m^3^, respectively [50]. However, the effects described in this research have been determined for a mean exposure of 19.7 μg/m^3^ NO_2_ over the entire pregnancy, which is merely half of the WHO annual guideline value. A recent meta-analysis has shown that exposure to increased levels of NO_2_ augments both respiratory and cardiovascular mortality and is in itself, apart from PM_2.5_, an important catalyst in disease development and even mortality [51]. These results thus show that the effects of NO_2_ exposure on the (micro-) circulation cannot be underestimated and should be further studied in terms of the effect of prenatal exposures on development later in life.

We acknowledge that this study has several strengths and limitations. A first strength is that this project is the first of its kind, investigating the effects of environmental PM_2.5_ and NO_2_ air pollution exposures occurring before birth, and during the child’s lifetime in both short- and long-term periods, on the microvasculature later in life. These results originate from a prospective birth cohort study, and thanks to extensive bio-banking, data collection at birth, and at follow-up examination, we were able to give a very precise estimation of the effect of exposure to air pollution on retinal vessel characteristics at the age of four to six. In this way, we contribute to the field of knowledge studying the complex relationships between prenatal and postnatal environmental exposures and (disease) development later in life. Secondly, we used data from a large population-based sample size of children, representative for the reproductive segment of the Flemish population of Belgium [26]. A third strength of this study is that retinal image analysis has been performed by one researcher blinded for the exposure data, excluding examiner bias. A limitation of this study is that although the confounding factors in our statistical model were selected following an a priori thorough examination, residual confounding posed by other environmental factors or population characteristics cannot be fully excluded. Another limitation is the potential misclassification of exposure. Our results are based on daily levels of residential particulate exposure during prenatal and postnatal life but do not account for exposures other than residential. However, the accuracy of our exposure models and relevance for personal and internal exposures have been proven, since air pollution levels at the residential address and proxies thereof, such as proximity of the home to major roads, correlate with the levels of nano-sized black carbon levels measured in the urine of children living in the same study area [34].

## Conclusions

Both prenatal and early childhood exposures to PM_2.5_ and NO_2_ were associated with changes in retinal vessel diameters and altered vessel tortuosity in young children. Our study adds to the knowledge of basic fundamental mechanisms on the complex relationship between early life exposure to ambient air pollution and cardiovascular disease development later in life. In future research projects, focus should be put on the implications of our findings on the cardiovascular development of the children in our prospective cohort.

## Supplementary information

**Additional file 1.**

## Data Availability

The datasets used and/or analyzed during the current study are available from the corresponding author on reasonable request.

## Abbreviations

BMI: Body mass index
CI: Confidence interval
CRAE: Central retinal arteriolar equivalent
CRVE: Central retinal venular equivalent
IQR: Interquartile range
MAP: Mean arterial pressure
NO_2_: Nitrogen dioxide
OD: Optic disk
PM: Particulate matter
PM_2.5_: Particulate matter with a diameter smaller than 2.5 μm
TI: Tortuosity index
