# Supplementary material for: Children’s microvascular traits and ambient air pollution exposure during pregnancy and early childhood: prospective evidence to elucidate the developmental origin of particle-induced disease
Source: BMC Med. 2020 May 26;18:128. doi: 10.1186/s12916-020-01586-x (PMC7249678; doi:10.1186/s12916-020-01586-x)
Supplement: Supplementary file 1 — Additional file 1. [file 12916_2020_1586_MOESM1_ESM.docx]

**ADDITIONAL FILE 1**

Children’s microvascular traits and air pollution exposure during pregnancy and early childhood: prospective evidence to elucidate the developmental origin of particle-induced disease

Leen J. Luyten^1,2^, Yinthe Dockx^1^, Eline B. Provost^1,3^, Narjes Madhloum^1^, Hanne Sleurs^1^, Kristof Y. Neven^1^, Bram G. Janssen^1^, Hannelore Bové^1^, Florence Debacq-Chainiaux^2^, Nele Gerrits^3^, Wouter Lefebvre^3^, Michelle Plusquin^1^, Charlotte Vanpoucke^4^, Patrick De Boever^1,3^, Tim S. Nawrot^1,5^

^1^Centre for Environmental Sciences, Hasselt University, Hasselt, Belgium;

^2^Unité de Recherche en Biologie Cellulaire (URBC) - Namur Research Institute for Life Sciences (Narilis), Namur University, Namur, Belgium;

^3^Health unit, Flemish Institute for Technological Research (VITO), Mol, Belgium;

^4^Belgian Interregional Environment Agency (IRCELINE), Brussels, Belgium.

^5^Department of Public Health & Primary Care, Occupational and Environmental Medicine, Leuven University, Leuven, Belgium.

**ADDITIONAL TABLES**

**Table S1. Comparison of characteristics of the eligible mother-child pairs who participated in the follow-up study and had analyzed retinal pictures and full data (n = 245) or had poor quality pictures or missing data (n = 87) or did not participate in the follow-up study of the ENVIR*ON*AGE birth cohort (n = 242).**

|  | Participants follow-up with analysed retina pictures and full data (n = 245) | Participants follow-up with poor quality retina pictures and/or missing data (n = 87) | Non-participants follow-up (n = 242) | Overall  p-value |
| --- | --- | --- | --- | --- |
| Characteristics |  |  |  |  |
| Mother |  |  |  |  |
| Age at birth child, years | 29.9 (4.1)^a^ | 30.5 (4.6) ^b^ | 27.9 (4.9) ^a,b^ | <0.0001 |
| Pre-pregnancy BMI, kg/m² | 24.4 (4.6) | 23.6 (4.2) ^a^ | 24.8 (4.8) ^a^ | 0.10 |
| Parity |  |  |  | 0.84 |
| 1 | 125 (51.0) | 51 (58.6) | 128 (52.9) |  |
| 2 | 92 (37.6) | 25 (28.7) | 90 (37.2) |  |
| 3 ≤ | 28 (11.4) | 11 (12.7) | 24 (9.9) |  |
| Smoking behavior during pregnancy |  |  |  | 0.10 |
| Never smoked | 167 (68.2) | 61 (70.1) | 140 (57.8) |  |
| Stopped smoking before pregnancy | 46 (18.8) | 17 (19.6) | 59 (24.4) |  |
| Smoked during pregnancy | 32 (13.0) | 9 (10.3) | 43 (17.8) |  |
| Education level |  |  |  | <0.0001 |
| Low (no high school diploma) | 16 (6.5) ^a^ | 6 (6.9) ^b^ | 49 (20.3) ^a,b^ |  |
| Middle (high school diploma) | 64 (26.1) | 33 (37.9) | 109 (45.0) |  |
| High (college degree or higher) | 165 (67.4) | 48 (55.2) | 84 (34.7) |  |
|  |  |  |  | *(Continued)* |

**Table S1. Continued**

|  | Participants follow-up with analysed retina pictures and full data (n = 245) | Participants follow-up with poor quality retina pictures and/or missing data (n = 87) | Non-participants follow-up  (n = 242) | Overall  p-value |
| --- | --- | --- | --- | --- |
| Child |  |  |  |  |
| Birth weight, g | 3446.6 (429.8) | 3447.1 (459.6) | 3446.2 (418.4) | 0.99 |
| Birth length, cm | 50.4 (1.9) | 50.6 (1.8) | 50.4 (1.9) | 0.76 |
| Ethnicity |  |  |  | <0.0001 |
| European | 230 (93.9) ^a^ | 82 (94.3) ^b^ | 197 (81.4) ^a,b^ |  |
| Non-European | 15 (6.1) | 5 (5.7) | 45 (18.6) |  |
| Sex |  |  |  | 0.54 |
| Male | 116 (47.3) | 47 (54.0) | 122 (50.4) |  |
| Female | 129 (52.7) | 40 (46.0) | 120 (49.6) |  |
|  |  |  |  |  |
| Exposure |  |  |  |  |
| PM_2.5_, µg/m³ |  |  |  |  |
| Pregnancy |  |  |  |  |
| Trimester 1 | 14.3 (5.5) | 13.0 (4.9) | 13.6 (5.0) | 0.10 |
| Trimester 2 | 14.3 (5.1) | 13.9 (5.2) | 13.6 (5.1) | 0.29 |
| Trimester 3 | 14.2 (5.7) | 13.9 (5.3) | 14.2 (5.3) | 0.89 |
| Entire pregnancy | 14.3 (2.3) ^a,b^ | 13.6 (2.5) ^a^ | 13.8 (2.4) ^b^ | 0.02 |
| Childhood |  |  |  |  |
| Average childhood exposure | 12.6 (1.1) | 12.6 (1.2) | 12.5 (1.3) | 0.54 |
|  |  |  |  | *(Continued)* |

**Table S1. Continued**

|  | Participants follow-up with analysed retina pictures and full data (n = 245) | Participants follow-up with poor quality retina pictures and/or missing data (n = 87) | Non-participants follow-up  (n = 242) | Overall  p-value |
| --- | --- | --- | --- | --- |
| NO_2_, µg/m³ |  |  |  |  |
| Pregnancy |  |  |  |  |
| Trimester 1 | 19.9 (6.0) | 18.6 (6.2) | 19.3 (5.6) | 0.21 |
| Trimester 2 | 19.8 (6.2) | 19.5 (6.1) | 19.4 (5.9) | 0.77 |
| Trimester 3 | 19.6 (6.2) | 19.1 (5.8) | 19.8 (6.2) | 0.62 |
| Entire pregnancy | 19.7 (4.4) | 19.1 (4.8) | 19.5 (4.2) | 0.46 |
| Childhood |  |  |  |  |
| Average childhood exposure | 17.2 (3.4) | 17.1 (3.3) | 17.5 (3.8) | 0.58 |

Groups with significant differences are indicated with the letters ‘a’ and ‘b’. Abbreviations: BMI, body mass index; SD, standard deviation.

**Table S2. Associations between either CRAE or CRVE and NO_2_ exposure during pregnancy, stratified for mean arterial pressure.**

|  |  | CRAE | | | CRVE | | | |
| --- | --- | --- | --- | --- | --- | --- | --- | --- |
|  | **Window of NO_2_ exposure during pregnancy** | **Change, µm** | **95% CI** | **p-value** | | **Change, µm** | **95% CI** | **p-value** |
| Low MAP | Entire pregnancy | 2.25 | -1.81 to 8.65 | 0.27 | | 3.41 | -1.84 to 8.65 | 0.20 |
|  |  |  |  |  | |  |  |  |
| High MAP | Entire pregnancy | 3.87 | 0.04 to 7.69 | 0.05 | | 4.71 | -0.72 to 10.13 | 0.09 |

The cut-off value for stratification of the MAP in a low and high group was determined by the median value of the study population, equalling 68.22 mmHg. Estimates are given as change in CRAE or CRVE and the according 95% confidence interval (CI) for every IQR increase in exposure to NO_2_ within the entire period of pregnancy. All models were adjusted for age (years), sex, ethnicity, mean arterial blood pressure and BMI of the child at the moment of the follow-up visit, the season in which the follow-up examination took place, birth weight (grams), maternal age at the birth of her child and pre-pregnancy BMI, maternal education level, alcohol use of the mother during pregnancy, smoking habits of the mother before and during pregnancy, and the exposure of the child to passive smoking. Abbreviations: CI, Confidence interval; CRAE, Central retinal venular equivalent; CRVE, Central retinal venular equivalent; MAP, mean arterial pressure; NO_2_, Nitrogen dioxide.

**Table S3. Sensitivity analysis excluding mothers with hypertension (n = 8) and gestational diabetes (n = 15).**

|  |  | CRAE | | CRVE | | TI | |
| --- | --- | --- | --- | --- | --- | --- | --- |
|  | **Window of exposure during pregnancy** | **Change, µm** | **95% CI** | **Change, µm** | **95% CI** | **Change** | **95% CI** |
| PM_2.5_ | Trimester 1 | 2.23 | -2.73 to 7.19 | 1.21 | -5.40 to 7.82 | 0.0014 | -0.0029 to 0.0057  . |
|  | Trimester 2 | 1.47 | -1.75 to 4.70 | 3.00 | -1.30 to 7.30 | 0.00003 | -0.0028 to 0.0028 |
|  | Trimester 3 | 3.69 | -1.19 to 8.58 | 2.65 | -3.86 to 9.15 | 0.0032 | -0.0011 to 0.0074 |
|  | Entire pregnancy | 2.71 | -0.14 to 5.57 | 3.41 | -0.40 to 7.21 | 0.0014 | -0.0011 to 0.0039 |
|  |  |  |  |  |  |  |  |
| NO_2_ | Trimester 1 | 0.51 | -3.02 to 4.05 | 1.48 | -3.22 to 6.17 | 0.0014 | -0.0017 to 0.0043 |
|  | Trimester 2 | 1.43 | -2.48 to 5.35 | 2.09 | -3.11 to 7.29 | -0.0006 | -0.0039 to 0.0028 |
|  | Trimester 3 | 1.42 | -2.26 to 5.10 | 1.61 | -3.27 to 6.50 | 0.0037* | 0.0006 to 0.0069 |
|  | Entire pregnancy | 2.54 | -0.18 to 5.27 | 3.68* | 0.06 to 7.31 | 0.0027* | 0.0004 to 0.0051 |

Estimates are given as change in CRAE, CRVE or TI and the according 95% confidence interval (CI) for every IQR increase in exposure to either PM_2.5_ or NO_2_ within the three trimesters and entire period of pregnancy. All models were adjusted for age (years), sex, ethnicity, mean arterial blood pressure and BMI of the child at the moment of the follow-up visit, the season in which the follow-up examination took place, birth weight (grams), maternal age at the birth of her child and pre-pregnancy BMI, maternal education level, alcohol use of the mother during pregnancy, smoking habits of the mother before and during pregnancy, and the exposure of the child to passive smoking. Models investigating trimester-specific exposure were adjusted for the three pregnancy trimester-averaged exposures levels. Abbreviations: CI, Confidence interval; CRAE, Central retinal venular equivalent; CRVE, Central retinal venular equivalent; NO_2_, Nitrogen dioxide; PM_2.5_, Particulate matter with a diameter smaller than 2.5 μm; TI, Tortuosity index. * (p ≤ 0.05).

**Table S4. Sensitivity analysis excluding participants born from premature pregnancies (gestational age lower than 37 weeks) (n = 12).**

|  |  | CRAE | | CRVE | | TI | |
| --- | --- | --- | --- | --- | --- | --- | --- |
|  | **Window of exposure during pregnancy** | **Change, µm** | **95% CI** | **Change, µm** | **95% CI** | **Change** | **95% CI** |
| PM_2.5_ | Trimester 1 | 1.67 | -3.12 to 6.45 | 2.28 | -4.22 to 8.78   \| -4.91 \| \| --- \| \| -1.51 \| \| -3.89 \| \| -0.58 \| | 0.0035 | -0.0006 to 0.0076 |
|  | Trimester 2 | 1.67 | -1.55 to 4.90 | 2.81 | -1.57 to 7.19 | 0.0002 | -0.0026 to 0.0030 |
|  | Trimester 3 | 3.17 | -1.69 to 8.02 | 3.55 | -3.04 to 10.14 | 0.0046* | 0.0004 to 0.0088 |
|  | Entire pregnancy | 2.58 | -0.29 to 5.46 | 3.56 | -0.34 to 7.74 | 0.0021 | -0.0004 to 0.0045 |
|  |  |  |  |  |  |  |  |
| NO_2_ | Trimester 1 | 0.62 | -2.80 to 4.04 | 2.41 | -2.22 to 7.04 | 0.0026 | -0.0003 to 0.0055 |
|  | Trimester 2 | 1.77 | -2.15 to 5.69 | 1.89 | -3.41 to 7.20 | -0.0011 | -0.0045 to 0.0022 |
|  | Trimester 3 | 1.23 | -2.40 to 4.86 | 2.10 | -2.80 to 7.01 | 0.0043* | 0.0012 to 0.0073 |
|  | Entire pregnancy | 2.74* | 0.02 to 5.47 | 4.35* | 0.67 to 8.04 | 0.0033* | 0.0010 to 0.0056 |

Estimates are given as change in CRAE, CRVE or TI and the according 95% confidence interval (CI) for every IQR increase in exposure to either PM_2.5_ or NO_2_ within the three trimesters and entire period of pregnancy. All models were adjusted for age (years), sex, ethnicity, mean arterial blood pressure and BMI of the child at the moment of the follow-up visit, the season in which the follow-up examination took place, birth weight (grams), maternal age at the birth of her child and pre-pregnancy BMI, maternal education level, alcohol use of the mother during pregnancy, smoking habits of the mother before and during pregnancy, and the exposure of the child to passive smoking. Models investigating trimester-specific exposure were adjusted for the three pregnancy trimester-averaged exposures levels. Abbreviations: CI, Confidence interval; CRAE, Central retinal venular equivalent; CRVE, Central retinal venular equivalent; NO_2_, Nitrogen dioxide; PM_2.5_, Particulate matter with a diameter smaller than 2.5 μm; TI, Tortuosity index. * (p ≤ 0.05).

**Table S5. Sensitivity analysis excluding participants who smoked during pregnancy (n = 32).**

|  |  | CRAE | | CRVE | | TI | |
| --- | --- | --- | --- | --- | --- | --- | --- |
|  | **Window of exposure during pregnancy** | **Change, µm** | **95% CI** | **Change, µm** | **95% CI** | **Change** | **95% CI** |
| PM_2.5_ | Trimester 1 | 3.02 | -1.95 to 7.98 | 4.60 | -2.25 to 11.45 | 0.0028 | -0.0016 to 0.0072 |
|  | Trimester 2 | 1.87 | -1.52 to 5.26 | 2.55 | -2.13 to 7.22 | 0.0002 | -0.0028 to 0.0032 |
|  | Trimester 3 | 3.64 | -1.23 to 8.52 | 4.04 | -2.68 to 10.77 | 0.0033 | -0.0010 to 0.0077 |
|  | Entire pregnancy | 3.10* | 0.13 to 6.08 | 3.92 | -0.18 to 8.02 | 0.0018 | -0.0008 to 0.0045 |
|  |  |  |  |  |  |  |  |
| NO_2_ | Trimester 1 | 1.70 | -1.97 to 5.38 | 4.41 | -0.63 to 9.45 | 0.0029 | -0.0003 to 0.0062 |
|  | Trimester 2 | 1.04 | -3.08 to 5.17 | 0.02 | -5.65 to 5.96 | -0.0013 | -0.0049 to 0.0023 |
|  | Trimester 3 | 1.32 | -2.42 to 5.06 | 2.11 | -3.02 to 7.25 | 0.0036* | 0.0003 to 0.0069 |
|  | Entire pregnancy | 2.79* | -0.002 to 5.58 | 3.85* | 0.002 to 7.69 | 0.0030* | 0.0005 to 0.0054 |

Estimates are given as change in CRAE, CRVE or TI and the according 95% confidence interval (CI) for every IQR increase in exposure to either PM_2.5_ or NO_2_ within the three trimesters and entire period of pregnancy. All models were adjusted for age (years), sex, ethnicity, mean arterial blood pressure and BMI of the child at the moment of the follow-up visit, the season in which the follow-up examination took place, birth weight (grams), maternal age at the birth of her child and pre-pregnancy BMI, maternal education level, alcohol use of the mother during pregnancy, smoking habits of the mother before and during pregnancy, and the exposure of the child to passive smoking. Models investigating trimester-specific exposure were adjusted for the three pregnancy trimester-averaged exposures levels. Abbreviations: CI, Confidence interval; CRAE, Central retinal venular equivalent; CRVE, Central retinal venular equivalent; NO_2_, Nitrogen dioxide; PM_2.5_, Particulate matter with a diameter smaller than 2.5 μm; TI, Tortuosity index. * (p ≤ 0.05).

**ADDITIONAL FIGURES**


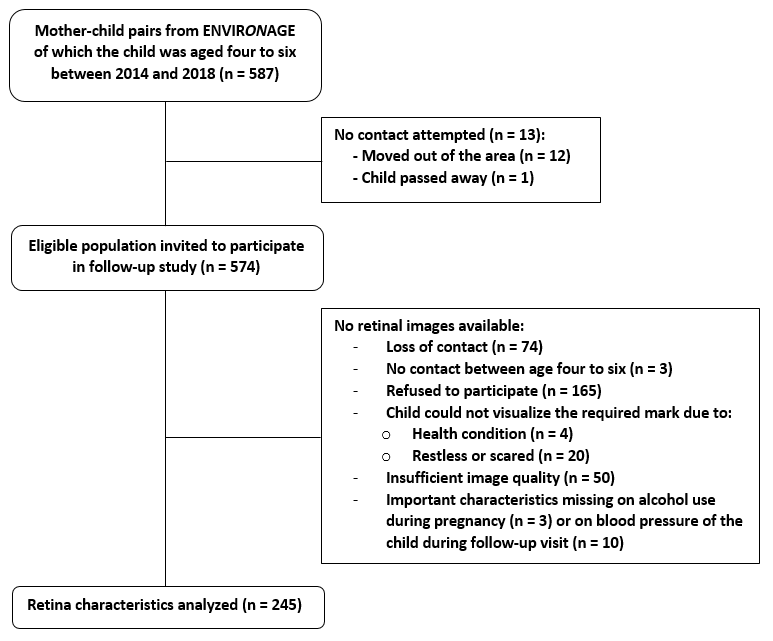


**Figure S1.** Flow chart of the selection process resulting in the 245 participants of this study.


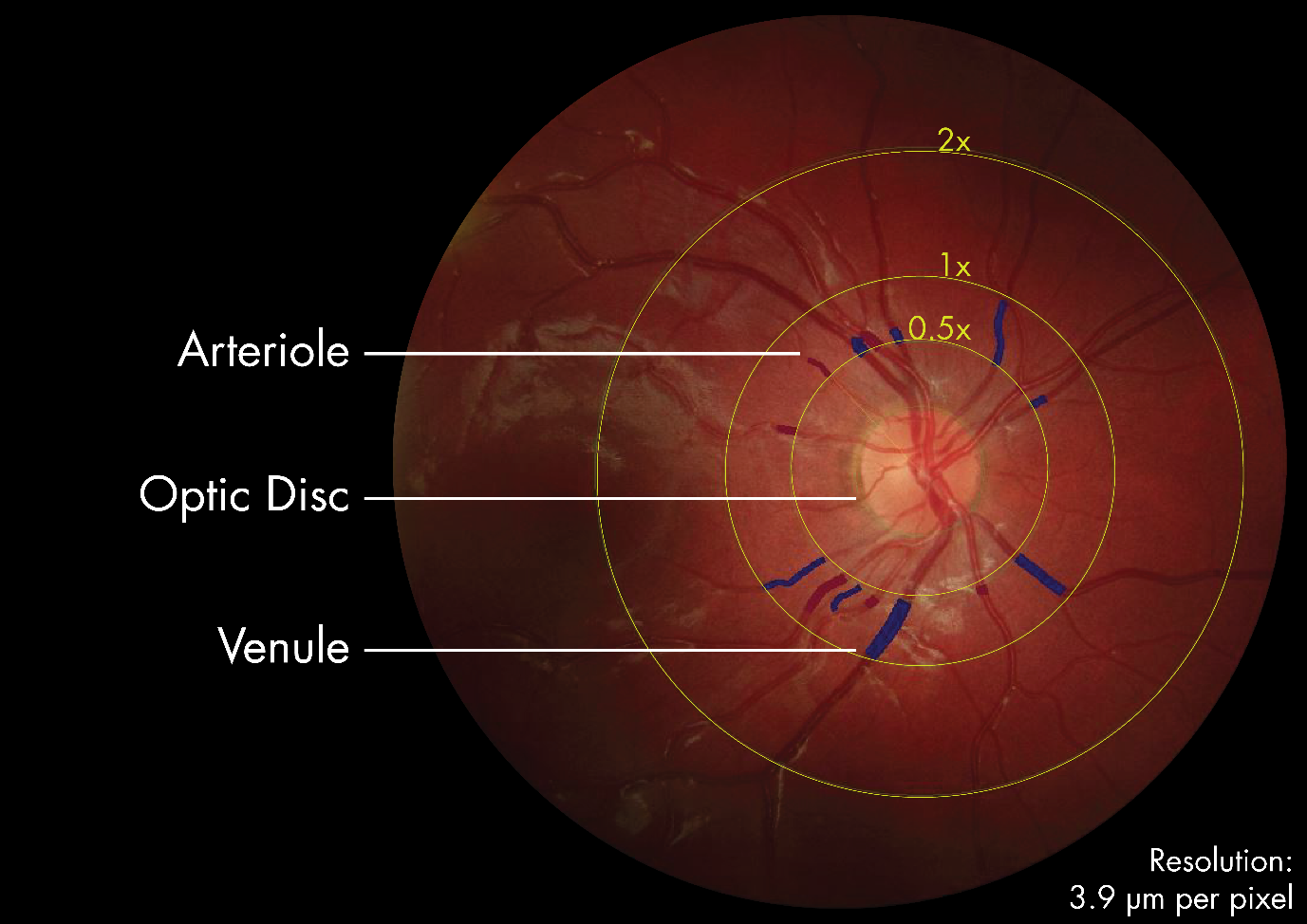


**Figure S2.** The central retinal arteriolar equivalent (CRAE) and central retinal venular equivalent (CRVE) were calculated within 0.5 and 1 times the diameter of the optic disc, starting from its margin. Arterioles within this area are indicated in red, the venules in blue.
